# Supplementary material for: Identification of photoperiod-induced specific miRNAs in the adrenal glands of Sunite sheep (Ovis aries)
Source: Front Vet Sci. 2022 Jul 22;9:888207. doi: 10.3389/fvets.2022.888207 (PMC9354845; doi:10.3389/fvets.2022.888207)
Supplement: Supplementary file 1 [file Data_Sheet_1.ZIP › Supplementary file/miRDeep2 file.zip!/miRDeep2 file/Know_Structure/LP42A3/oar-mir-379.pdf]

miRBase precursor : oar-mir-379
Total read count : 59186
oar-miR-379-5p read count : 55818
oar-miR-379-3p read count : 3366
remaining reads : 2

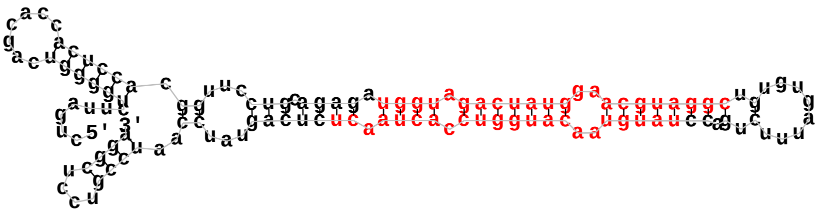

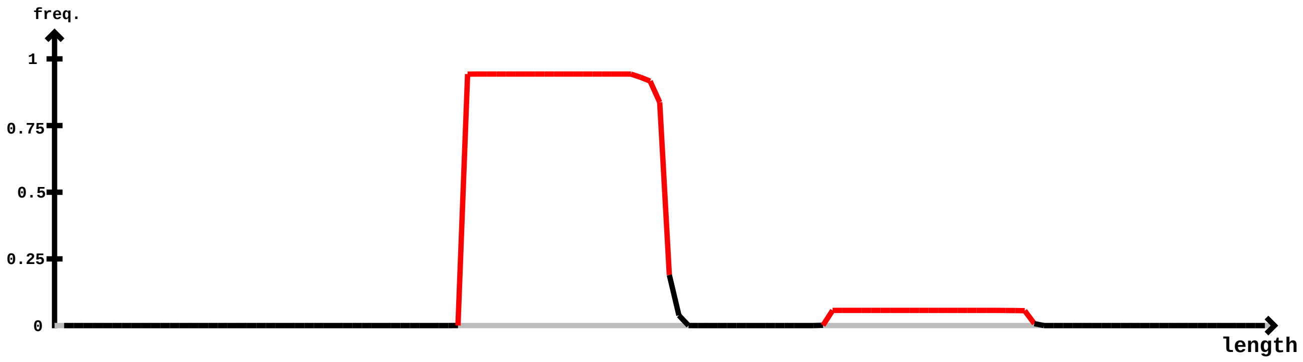

oar-miR-379-3p

oar-miR-379-5p

```
5'- cugauucuggggucagcaccacuccacgguuccugcagagaugguagacuauggaacguaggcugugugauuucugaccuauguaacaugguccacuaacucucaguauccaauccguccucgga   -3'   exp
    .......((((((.......)))))).((...(((.((((.((((.(((((((..((((((((.(........).).)))))))..))))))).)))).)))))))...))..((((....))))   reads   mm
    .....................cuccacgguuccugcagaga....................................................................................   1       0
    ......................uccacgguuccugcagaga....................................................................................   1       0
    ........................................augguagacuauggaacgua.................................................................   1       0
    ........................................augguagacuauggaacguagg...............................................................   9       0
    .........................................ugguagacuauggaacg...................................................................   3       0
    .........................................ugguagacuauggaacgu..................................................................   705     0
    .........................................ugguagacuauggaacgua.................................................................   851     0
    .........................................ugguagacuauggaacguag................................................................   4703    0
    .........................................ugguagacuauggaacguagg...............................................................   38288   0
    .........................................ugguagacuauggaacguaggc..............................................................   8973    0
    .........................................ugguagacuauggaacguaggcu.............................................................   2260    0
    .........................................ugguagacuauggaacguaggcug............................................................   6       0
    .........................................ugguagacuauggaacguaggcugu...........................................................   2       0
    ..........................................gguagacuauggaacguag................................................................   1       0
    ..........................................gguagacuauggaacguagg...............................................................   14      0
    ..........................................gguagacuauggaacguaggc..............................................................   2       0
    .............................................................................ccuauguaacaugguccacuaacu........................   1       0
    ..............................................................................cuauguaacaugguccacu............................   3       0
    ..............................................................................cuauguaacaugguccacC............................   1       1
    ..............................................................................cuauguaacaugguccacua...........................   2       0
    ..............................................................................cuauguaacaugguccacuaac.........................   54      0
    ..............................................................................cuauguaacaugguccacuaacu........................   3       0
    ...............................................................................uauguaacaugguccacA............................   1       1
    ...............................................................................uauguaacaugguccacC............................   1       1
    ...............................................................................uauguaacaugguccacu............................   10      0
    ...............................................................................uauguaacaugguccacua...........................   16      0
    ...............................................................................uauguaacaugguccacuaa..........................   34      0
    ...............................................................................uauguaacaugguccacuaG..........................   3       1
    ...............................................................................uauguaacaugguccacuaac.........................   2804    0
    ...............................................................................uauguaacaugguccacuaacu........................   427     0
    ...............................................................................uauguaacaugguccacuaacuc.......................   1       0
    ................................................................................auguaacaugguccacuaac.........................   3       0
    ................................................................................auguaacaugguccacuaacu........................   2       0
```
